# Fluconazole and echinocandin resistance of *Candida* species in invasive candidiasis at a university hospital during pre-COVID-19 and the COVID-19 outbreak

**DOI:** 10.1017/S0950268823001346

**Published:** 2023-08-25

**Authors:** Jidapa Szekely, Wiraphan Rakchang, Paramaporn Rattanaphan, Narongdet Kositpantawong

**Affiliations:** 1 Faculty of Medical Technology, Prince of Songkla University, Hat Yai, Thailand; 2 Department of Pathology, Faculty of Medicine, Ramathibodi Hospital, Mahidol University, Bangkok, Thailand; 3 Clinical Microbiology Unit, Department of Pathology, Faculty of Medicine, Prince of Songkla University, Hat Yai, Thailand; 4 Department of Internal Medicine, Faculty of Medicine, Prince of Songkla University, Hat Yai, Thailand

**Keywords:** azole-resistant, *Candida*, COVID-19, echinocandin-resistant, non-albicans

## Abstract

Antifungal susceptibility of *Candida* species is decreasing. Successful treatment for antifungal-resistant candida infection is challenging and associated with significant mortality. We performed a prospective observational study to identify the species and antifungal susceptibilities of invasive isolates of *Candida* species over a 5-year period at a university hospital in southern Thailand. Between 2017 and 2021, the species distribution was 39.1% *Candida tropicalis*, 24.8% *Candida albicans*, 20.3% *Candida parapsilosis* complex, 10.5% *Candida glabrata*, and 5.2% miscellaneous *Candida* spp. Notable observations include elevated minimal inhibitory concentration (MIC) and decrease susceptibility of *C. tropicalis* and *C. glabrata* to echinocandin and all tested triazoles. A shift of MIC_90_ value in the COVID-19 era was seen in *C. albicans* and *C. tropicalis* with azoles and echinocandins. Azole resistance increased among *C. tropicalis* isolates, and echinocandin resistance also increased among *C. parapsilosis* and *C. glabrata* isolates. Novel alterations in *FKS*1 HS1 and HS2 were detected in both isolates of anidulafungin-resistant *C. parapsilosis.* As *Candida* species have become more resistant to azoles and less susceptible to echinocandin development, the need arose to observe the emergence of resistance to both antifungal classes in candida clinical isolates, for a more effective infection control in the hospital.

## Introduction

The World Health Organization (WHO) released the first-ever list of priority fungal pathogens, considering research needs and perceived public health importance. Of the 19 listed pathogens, the four most common *Candida* species (*Candida albicans, Candida glabrata, Candida tropicalis,* and *Candida parapsilosis*) are focused on and categorised by WHO into critical and high-prioritised groups, due to candida’s ability of invasive acute and subacute systemic infection and the emerging of antifungal resistance in these species [1]. Invasive candida infection in the deep tissues and internal organs is a significant cause of death in numerous immunocompromised patients. More than 15 *Candida* species have been described as aetiologic agents of invasive candidiasis, and more than 90% of cases are attributed to five species: *C. albicans, C. glabrata, C. parapsilosis, C. tropicalis*, and *Candida krusei* [2]. In the United States and the Asia Pacific region, *C. albicans* has decreased in proportion significantly, accounting for less than 50% of all candida infections. Conversely, non-albicans *Candida* with decreased susceptibility to antifungals is dramatically increasing.

Species distribution varies depending on geographic regions and site of infections. In the United States, *C. parapsilosis* species complex (SC) is the predominant causative agent (41%), followed by *C. albicans* (37%), *C. glabrata* SC (10%), *C. tropicalis* (7%), *C. krusei* (1%), and other rare *Candida* spp. (4%) [3]. *C. parapsilosis* SC is the most common cause of non-albicans bloodstream infection and is a significant pathogen in neonates [4]. *C. glabrata* causes systemic infections in HIV and immune-suppressed patients [5–7]. *C. tropicalis* is commonly associated with a high mortality rate in patients with neutropenia and malignancy [8, 9]. *C. krusei* is a frequent cause of fungemia in patients with haematologic malignant neoplasms [10]. Notably, changing candida epidemiology, especially of non-albicans species, may impact on changing trends in antifungal susceptibility patterns and treatment options for candida infections [11].

Two important tools to detect antifungal drug resistance/susceptibility and to guide patient therapy are species identification and antifungal susceptibility testing (AFST). The minimal inhibitory concentration (MIC) of drug required to inhibit the organisms *in vivo* is normally determined by *in vitro* testing with live pathogens isolated from patients using a broth dilution technique. In Thailand, in the past decade, AFST has been available for routine clinical laboratory use only in tertiary care, and university hospitals. Standardised methods, up-to-date interpretive criteria, and clear clinical interpretations are needed for interlaboratory comparison and determining drug resistance trends nationally and globally. The lack of sufficient antifungal susceptibility data exacerbates the difficulties of dealing with drug resistant pathogens and clinical management, even when proper identification of the suspected pathogens is provided. Therefore, AFST data are valuable at both the institutional and international levels [12].

The emergence of drug-resistant candida has posed additional challenges to successful treatment. The selection of appropriate antifungal agents for candida infection treatment has become more complex and difficult and requires careful consideration of the various outbreaks of antifungal resistance. One prominent example was found by the SENTRY antifungal surveillance programme run from 2006 to 2016 in Asia-Pacific countries. Fluconazole resistance was found in all four common *Candida* species: *C. albicans, C. tropicalis*, *C. parapsilosis,* and *C. glabrata*, and the programme documented a lower percentage of therapeutic and empirical treatment success. Increased MIC values were also associated with the reduction of treatment outcomes. Treatment success rates for candida resistant to fluconazole [13], voriconazole [14], and itraconazole [15] were 37%, 38%, and 67%, respectively. Besides azole resistance, another significant concern is echinocandin resistance in candida. The most common echinocandin-resistant strains were *C. glabrata* with high rates (10%) at studied institutions. Resistance is commonly detected after 3–4 weeks of treatment and is associated with a poor outcome [16]. Updated distribution of *Candida* species and antifungal drug susceptibility profiles of isolates from patients receiving antifungal therapy is therefore important for surveillance studies. In addition, study of drug resistance mechanisms, for example, mutations of genes involved in drug targets, can provide useful data on trends of candida in hospitals, particularly for efficient monitoring of drug-resistant outbreaks in the future.

The echinocandin-resistant mechanism in candida has been described as the result of a mutation in a gene related to the 1,3-beta-D-glucan synthase complex, an enzyme crucial for cell wall biosynthesis localised to the plasma membrane [17]. Studies have also identified point mutations in two common locations, that is, the highly conserved ‘hotspots’ (HSs) of HS1 and HS2 of the *FKS1* gene, a key gene encoding the catalytic subunit of 1,3-beta-D-glucan synthase [18]. Resistance through this mechanism has been reported in *C. albicans* and non-albicans *Candida* species, including *C. glabrata, C. krusei, C. tropicalis,* and *Candida dubliniensis.* In addition, reduction of echinocandin susceptibility in *C. glabrata* was also affected by mutations in the *FKS2* gene [19]. The point mutations in the *FKS1* and *FKS2* genes spontaneously arise in the presence of echinocandin selection pressure in *C. glabrata.* Such mutations in either of these genes result in amino acid substitutions in the glucan synthase, leading to decreased susceptibility with an increase in the MIC to echinocandin.

Following the COVID-19 pandemic, the incidence of invasive candidiasis increased due to the complicated nature and severe clinical course of many COVID-19 infections. Recently, a decrease in susceptibility to antifungals was observed in some invasive isolates with, in particular, a marked increase of resistance to fluconazole and voriconazole – among *C. tropicalis* and *C. parapsilosis* during the COVID-19 period. In contrast, the rate of fluconazole-resistant *C. glabrata* decreased. Since COVID-19-related data of candida susceptibility are scarce, we aimed to compare the prevalence of drug-resistant candida isolates before and during the pandemic [20]. Here, we report the surveillance results of clinically important *Candida* species over a 5-year period (January 2017 to December 2021; pre- and during COVID-19 periods) recovered from patient’s specimens, and their rates of resistance to antifungal drugs expressed as MIC_50_ and MIC_90_ values. A novel alteration in the *FKS1* gene in echinocandin-resistant *C. parapsilosis* was documented in the study.

## Methods

### Organisms

**Figure 1.**
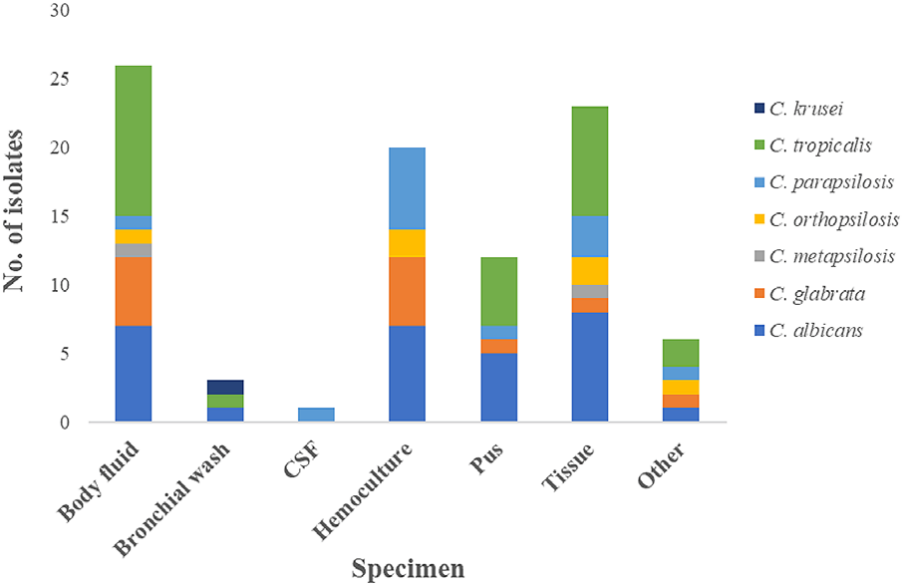
Distribution of candida isolates from each specimen.

In total, 133 isolates of *Candida* spp. were isolated from specimens – blood, tissues, and body fluids – taken from inpatients admitted during the study period. No repeated isolates from the same patient were collected. The study protocol was conducted in accordance with the Declaration of Helsinki and Good Clinical Practice principles. Ethical approval (REC 58-371-19-2) was granted by the Human Research Ethic Committee, Faculty of Medicine, Prince of Songkla University (Songkhla, Thailand). Isolates were identified to the species level using standard biochemical tests (sugar assimilation and fermentation, and structural morphology, i.e., germ tube and chlamydoconidia formation). Species identification was confirmed by matrix-assisted laser desorption/ionisation–time-of-flight mass spectrometry analysis as per the manufacturer’s instructions (Bruker Microflex LT, Bruker Daltonik GmbH, Bremen, Germany). The species identity of selected organisms was assigned without specific patient information. Species confirmation was verified by sequence analysis of the internal transcribed spacer region against online databases. The species distribution of isolates is shown in Figure 1.

### Antifungal susceptibility testing and interpretation

The collection of 133 isolates comprised 52 isolates of *C. tropicalis*, 32 of *C. albicans*, 27 of *C. parapsilosis* complex, 14 of *C. glabrata*, and 1 isolate each of *C. krusei, Candida guilliermondii, Candida caribbica, Candida fabianii, Candida duobushaemulonii, Candida rugosa*, and *Candida nivariensis.* The Sensititre YeastOne colorimetric antifungal susceptibility test was performed using the protocol specified by the manufacturer (TREK Diagnostic Systems Ltd., West Sussex, UK). The culture was incubated to a turbidity of 0.5 McFarland standard, equating to a concentration range of 1 × 10^6^ to 5 × 10^6^ cells/ml. The turbidity-adjusted culture was transferred into the inoculum broth to achieve a final culture concentration of 1.5 × 10^3^ to 8 × 10^3^ cells/ml and added to the wells of the Sensititre plate containing antifungal drugs at various concentrations. Plates were incubated at 35°C and examined after 24 h. The MIC was recorded as the lowest concentration of the drug under test to inhibit visible growth of the culture. *C. parapsilosis* ATCC 22019 was used as the control strain. All tests were performed in duplicate, with interpretations of susceptibility of each isolate/drug combination based on the revised CLSI clinical breakpoint [21, 22]. These breakpoints were interpreted for *C. albicans*, *C. glabrata*, *C. parapsilosis* complex, *C. tropicalis*, and *C. krusei.* Epidemiological cutoff values (ECVs), if available, were applied for other species tested.

### DNA extraction, PCR amplification, and sequencing

Fungal nucleic acids were extracted using DNeasy Blood and Tissue kits (Qiagen, Hilden, Germany) according to the manufacturer’s instructions. To confirm the species of candida isolates, the internal transcribed spacer region 1 (ITS1) and/or ITS2 regions of rDNA genes [23] were amplified by PCR with ITS1 forward, and ITS4 universal reverse primer as described previously [24]. The *FKS* HS mutations of the *FKS* genes encoding the 1,3-β-D-glucan synthase subunits were screened by PCR amplification and sequencing using specific primers [25–27]. PCR fragments were amplified using the UCP HiFidelity PCR Kit (Qiagen) and cleaned with a PCR purification kit (Qiagen). *FKS*-HS sequencing of *C. glabrata* and *C. parapsilosis* isolates was performed as previously described [28, 29].

### Statistical analysis of clinical data

Electronic medical records were reviewed for patients with candidemia and invasive candidiasis. Data were obtained for baseline characteristics, underlying diseases, associated risk factors, type of candida infection, Acute Physiology and Chronic Health Evaluation II score, time of onset of candidemia after hospitalisation, prior fluconazole prescription, empiric antifungal treatment, and outcome. Patients were divided into fluconazole susceptible, and fluconazole dose-dependent susceptible group, and a fluconazole resistant group. Categorical variables are presented in both number and percent values. Continuous variables are expressed as mean (standard deviation) in normal distribution and median (interquartile range (IQR)) in abnormal distribution. Fisher’s exact test or Chi square were used for categorical variables, and Student’s *t-*test or Mann–Whitney *U* test for continuous variables. Calculation was performed with R software version 3.3.2 (R Foundation, Vienna, Austria), and a *P*-value of <0.05 was considered statistically significant.

## Results

### Distribution of candida isolates in clinical specimens

**Figure 2.**
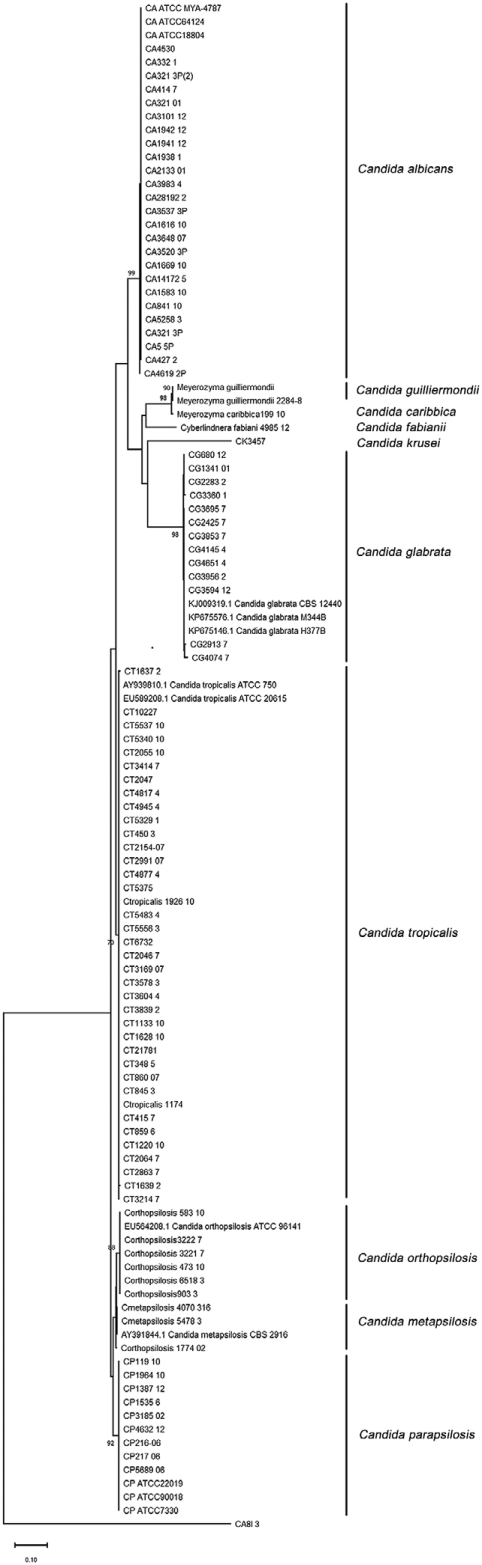
Phylogenetic tree of the internal transcribed spacer (ITS) region of rRNA gene of candida isolates identified in this study. ITS sequence tree was constructed with the Neighbour-Joining method and the Kimura-2 correction. Numbers appearing on each node represent bootstrap percentage after statistical analysis from 10,000 trees.

Candida isolates were recovered from blood (39.8%), body fluids (21.1%), tissue biopsies (17.2%), catheter urine (9.4%), aspirated pus (9.4%), bronchial wash (2.3%), and cerebrospinal fluid (0.8%). The distribution of the most prevalent species recovered from clinical samples and their source is shown in Figure 1. All isolates were identified by sequence-based analysis (Figure 2) and ranked according to their frequency: *C. tropicalis* (39.1%), *C. albicans* (24.8%), *C. parapsilosis* complex (20.3%), *C. glabrata* (10.5%), and miscellaneous (the latter including *C. krusei, C. guilliermondii, C. caribbica, C. fabianii, C. duobushaemulonii, C. rugosa,* and *C. nivariensis*).

*C. tropicalis* (47.1%) and *C. parapsilosis* (21.6%) were most frequently isolated from blood cultures, whereas *C. tropicalis* and *C. albicans* (41.7% and 36.4%, respectively) were the most common in tissue and pus specimens, and also from urinary tract infections (25.0% and 41.6%, respectively).

### MIC distributions of antifungal against Candida species between 2017 and 2021

The MIC range of nine agents tested against *Candida* species isolates are shown in Table 1. For species with published clinical breakpoints or ECVs, their percentage of susceptible or percentage of wild type (WT), respectively, were calculated. Notable observations include elevated MIC values and significantly decreased susceptibility of *C. albicans* and *C. tropicalis* to all tested azole during the COVID-19 period compared to pre-COVID-19, *C. tropicalis.* Moreover, *C. glabrata* displayed low susceptibility to echinocandin, posaconazole, and voriconazole during the pre-COVID-19 period. Within the *C. parapsilosis* complex, *C. parapsilosis* showed a lower percentage of susceptibility to echinocandin and fluconazole than *Candida orthopsilosis* and *Candida metapsilosis.* The majority of *C. albicans* (>90%) and the uncommon *Candida* species were highly susceptible to echinocandin.

**Table 1.** Minimum inhibitory concentration (MIC) of tested *Candida* species to antifungal drugs during 2017–2021

Species	MIC range (μg/ml) (%Susceptible or %WT^a^)								
	AND	MF	CAS	5-FC	PZ	VOR	IZ	FZ	AB
*C. albicans*									
Pre-COVID-19 (2017–2019) (*n* = 27)	0.03–0.25	0.008–0.03	0.03–0.25	0.06–0.5	0.008–0.12	0.008–0.12	0.03–0.12	0.12–1	0.12–1
	(100)	(100)	(100)		(96.3)	(100)		(100)	(100)
COVID-19 (2020–2021) (*n* = 6)	0.03–0.12	0.008–0.5	0.03–0.25	0.06–0.5	0.03–8	0.008–8	0.06–16	0.12–256	0.25–0.5
	(100)	(83.3)	(100)		(50)	(50)		(66.7)	(100)
2017–2021 (*n* = 33)	0.03–0.25	0.008–0.5	0.03–0.25	0.06–0.5	0.008–8	0.008–8	0.03–0.16	0.12–256	0.12–1
	(100)	(97)	(100)		(96.3)	(90.9)		(93.9)	(100)
*C. glabrata*									
Pre-COVID-19 (*n* = 13)	0.015–2	0.008–4	0.06–8	0.06	0.008–2	0.008–1	0.15–4	0.12–32	1–4
	(69.2)	(76.9)	(46.1)		(69.2)	(30.7)	(100)	(0)	(92.3)
COVID-19 (*n* = 1)	0.03	0.03	0.03	0.06	1	0.25	0.5	8	1
	(100)	(100)	(100)		(100)	(100)	(100)	(0)	(100)
2017–2021 (*n* = 14)	0.015–2	0.008–4	0.03–8	0.06	0.008–2	0.008–1	0.15–4	0.12–32	1–4
	(71.4)	(78.6)	(50)		(71.4)	(35.7)	(100)	(0)	(92.9)
*C. parapsilosis*									
Pre-COVID-19 (*n* = 12)	0.5–8	0.5–4	0.5–1	0.06–0.25	0.03–0.25	0.015–0.25	0.06–0.25	0.05–8	0.25–1
	(83.3)	(83.3)	(100)		(100)	(91.6)	(100)	(75)	(100)
COVID-19 (*n* = 6)	1–2	1–2	0.5–1	0.06–64	0.03–0.12	0.008–0.06	0.06–0.25	0.25–4	0.25–0.5
	(100)	(100)	(100)		(100)	(100)	(100)	(66.7)	(100)
2017–2021 (*n* = 18)	0.5–8	0.5–4	0.5–1	0.06–64	0.03–0.25	0.008–0.25	0.06–0.25	0.25–8	0.25–1
	(88.9)	(88.9)	(100)		(100)	(94.4)	(100)	(72.2)	(100)
*C. orthopsilosis*									
Pre-COVID-19 (*n* = 7)	0.5–2	0.5–1	0.25–1	0.06–64	0.03–0.06	0.015	0.06–0.12	0.05–0.5	0.12–1
	(100)	(100)	(100)		(100)	(100)	(100)	(100)	(100)
*C. metapsilosis*									
Pre-COVID-19 (*n* = 2)	0.5–1	0.5	0.5	0.12–0.25	0.06	0.06	0.12	2	1
	(100)	(100)	(100)		(100)	(100)	(100)	(100)	(100)
*C. tropicalis*									
Pre-COVID-19 (*n* = 40)	0.03–0.12	0.015–0.08	0.03–0.5	0.06–0.25	0.06–8	0.06–8	0.12–16	0.5–256	0.25–2
	(100)	(100)	(85)		(48.5)	(80)	(89.7)	(84.6)	(100)
COVID-19 (*n* = 12)	0.12–0.5	0.03–0.25	0.03–0.5	0.06–0.25	0.12–8	0.12–8	0.12–16	1–256	0.5–1
	(91.7)	(100)	(91.7)		(16.7)	(16.7)	(66.7)	(41.7)	(100)
2017–2021 (*n* = 52)	0.03–0.5	0.015–0.12	0.03–0.5	0.06–0.25	0.06–8	0.06–8	0.12–16	0.5–256	0.25–2
	(98.1)	(100)	(86.5)		(34.6)	(57.7)	(82.7)	(73.1)	(100)
*C. duobushaemulonii* COVID-19 (*n* = 1)	0.25	0.06	0.06	0.06	0.12	0.12	0.25	8	2
	(100)	(100)	(100)		(100)	(100)	(100)	(100)	
*C. caribbica* Pre-COVID-19 (*n* = 1)^c^	1	0.5	0.5	0.06	0.25	0.12	0.5	4	0.5
*C. fabianii* Pre-COVID-19 (*n* = 1)^c^	0.03	0.03	0.03	0.06	0.25	0.03	0.12	2	0.12
*C. guilliermondii* Pre-COVID-19 (*n* = 1)	2	1	0.25	0.06	0.25	0.12	0.5	4	0.12
	(100)	(100)	(100)				(100)	(100)	(100)
*C. krusei* Pre-COVID-19 (*n* = 1)	0.12	0.12	0.5	16	0.25	0.5	0.25	32^b^	1
	(100)	(100)	(0)		(100)	(100)	(100)		(100)
*Candia nivariensis* COVID-19 (*n* = 1)^c^	0.015	0.008	0.12	0.5	0.03	0.015	1	16	1
*C. rugosa* COVID-19 (*n* = 1)^c^	0.06	0.03	0.5	0.06	0.03	0.015	0.03	2	1

5-FC, 5-flucytosine; AB, amphotericin B; AND, anidulafungin; Cas, caspofungin; FZ, fluconazole; IZ, itraconazole; MF, micafungin; PZ, posaconazole; VOR, voriconazole; WT, wild type.

a Wild type.

b Intrinsic resistant to fluconazole.

c No clinical breakpoint or epidemiological cutoff values.

### Susceptibility among candida isolates from the pre-COVID-19 and COVID-19 periods

Among the four most frequent *Candida* species during the COVID-19 period, MIC_90_ values of *C. albicans* were elevated for micafungin (0.03 μg/ml, pre-COVID-19 to 0.5 μg/ml, COVID-19), caspofungin (0.12 μg/ml, pre-COVID-19 to 0.25 μg/ml, COVID-19) and markedly so for all tested triazole compounds (Table 2). Resistance to all echinocandins (23.1%) was noted among *C. glabrata* in both sampling periods.

**Table 2.** Minimum inhibitory concentration (MIC_50_ and MIC_90_) of tested *Candida* species to antifungal drugs during 2017–2021

		CLSI 2017–2021			ECV 2017–2021		2017–2019 (pre-COVID-19)		2020–2021 (COVID-19)		2017–2021	
Species (no. of isolates)	Antifungal name	%S	%SDD/I^a^	%R	%WT^b^	%Non-WT	MIC_50_ (μg/ml)	MIC_90_ (μg/ml)	MIC_50_ (μg/ml)	MIC_90_ (μg/ml)	MIC_50_ (μg/ml)	MIC_90_ (μg/ml)
*C. albicans*							*n* = 27		*n* = 6		*n* = 33	
	Anidulafungin	100	0	0			0.06	0.12	0.12	0.12	0.12	0.12
	Micafungin	97	3	0			0.015	0.03	0.015	0.5	0.015	0.03
	Caspofungin	100	0	0			0.06	0.12	0.06	0.25	0.06	0.12
	5-Flucytosine	ND	ND	ND			0.12	0.5	0.12	0.25	0.12	0.5
	Posaconazole	ND	ND	ND	87.8	12.2	0.03	0.03	0.03	8	0.03	0.03
	Voriconazole	90.9	3	6.1			0.008	0.03	0.008	8	0.008	0.03
	Itraconazole	93.9	0	6.1			0.06	0.12	0.06	16	0.06	0.12
	Fluconazole	93.9	0	6.1			0.25	0.5	0.5	256	0.25	1
	AmphotericinB	ND	ND	ND	100	0	1	1	0.5	0.5	1	1
*C. glabrata*							*n* = 13		*n* = 1		*n* = 14	
	Anidulafungin	71.4	7.2	21.4			0.06	2	ND	ND	0.06	2
	Micafungin	78.6	0	21.4			0.015	4	ND	ND	0.015	4
	Caspofungin	50	28.6	21.4			0.25	8	ND	ND	0.12	8
	5-Flucytosine	ND	ND	ND			0.06	0.06	ND	ND	0.06	0.06
	Posaconazole	ND	ND	ND	71.4	28.6	1	2	ND	ND	1	2
	Voriconazole	ND	ND	ND	35.7	64.3	0.5	0.5	ND	ND	0.5	1
	Itraconazole	ND	ND	ND	100	0	1	1	ND	ND	0.5	4
	Fluconazole	0	100	0			16	32	ND	ND	16	32
	AmphotericinB	ND	ND	ND	92.9	7.1	1	2	ND	ND	1	4
*C. parapsilosis*							*n* = 12		*n* = 6		*n* = 18	
	Anidulafungin	88.9	0	11.1			1	8	2	2	2	8
	Micafungin	88.9	11.1	0			1	4	2	2	1	4
	Caspofungin	100	0	0			0.5	1	0.5	1	0.5	1
	5-Flucytosine	ND	ND	ND			0.12	0.25	0.06	64	0.12	0.25
	Posaconazole	ND	ND	ND	100	0	0.06	0.12	0.03	0.12	0.06	0.12
	Voriconazole	94.4	5.6	0			0.03	0.12	0.015	0.06	0.03	0.12
	Itraconazole	ND	ND	ND	100	0	0.12	0.25	0.06	0.25	0.12	0.25
	Fluconazole	72.2	16.7	11.1			2	8	0.5	4	1	8
	AmphotericinB	ND	ND	ND	100	0	0.5	1	0.5	0.5	0.5	1
*C. tropicalis*							*n* = 40		*n* = 12		*n* = 52	
	Anidulafungin	98.1	1.9	0			0.12	0.12	0.25	0.25	0.12	0.25
	Micafungin	100	0	0			0.03	0.06	0.06	0.06	0.03	0.06
	Caspofungin	86.5	13.5	0			0.12	0.5	0.12	0.25	0.12	0.5
	5-Flucytosine	ND	ND	ND			0.06	0.12	0.12	0.25	0.06	0.12
	Posaconazole	ND	ND	ND	34.6	65.4	0.25	1	0.5	8	0.25	8
	Voriconazole	57.7	25	17.3			0.12	8	0.25	8	0.12	8
	Itraconazole	ND	ND	ND	82.7	17.3	0.25	0.5	0.5	16	0.25	16
	Fluconazole	73.1	7.7	19.2			1	128	4	256	2	256
	AmphotericinB	ND	ND	ND	100	0	1	1	1	1	1	1

5-Flucytosine, posaconazole, and amphotericin B have no interpretation criteria according to CLSI guidelines. ND, not determined; S, susceptible; R, resistant.

a SDD/I, susceptible dose-dependent/intermediate.

b WT, wild type, interpretation for antifungal susceptibility with no clinical breakpoint.

### Screening for FKS mutation for echinocandin-resistant Candida species

Mutations in HS regions of the 1,3-β-D-GS-encoding genes were screened in isolates displaying MIC values above the clinical breakpoints of echinocandin agents (*C. glabrata*; 3/13 and *C. parapsilosis*; (2/12 Other *Candida* species did not exhibit echinocandin resistance with the exception of a single *C. albicans* isolate showing non-susceptibility to micafungin (MIC, 0.5 μg/ml). Three of five *C. glabrata* isolates were resistant to the echinocandins and exhibited a mutation encoding an *FKS2* HS1 alteration S663P (Table 3), whereas the two susceptible isolates were similar to WT controls. Two *C. parapsilosis* isolates displayed elevated MICs to anidulafungin (8 μg/ml) and micafungin (4 μg/ml) and showed a double mutation on *FKS1* HS1 V595I/*FKS1* HS2 Q1392H. A separate mutation at *FKS2* HS1 H501Q, in one of these isolates which was also fluconazole-resistant (MIC, 8 μg/ml). However, the mutation at *FKS2* HS1 S658L present in both echinocandin-resistant and echinocandin-susceptible isolates, possibly suggests that this alteration might not have a cumulative effect.

**Table 3.** Summary of *FKS* genes, 1,3-β-D-glucan synthase alterations in echinocandin-resistant *Candida glabrata* and *Candida parapsilosis*

		MIC, μg/ml			FKS1		FKS2	
Species	Specimen	ANF	CSF	MCF	HS1	HS2	HS1	HS2
*C. glabrata*								
CG3594	Blood	2 (R)	8 (R)	4 (R)	WT	WT	S663P	WT
CG1341	Tissue	2 (R)	8 (R)	2 (R)	WT	WT	S663P	WT
CG3360	Blood	2 (R)	8 (R)	4 (R)	WT	WT	S663P	WT
CG2285	Blood	0.03 (S)	0.06 (S)	0.015 (S)	WT	WT	WT	WT
CG680	Blood	0.015 (S)	0.06 (S)	0.008 (S)	WT	WT	WT	WT
*C. parapsilosis*								
CP5216	Blood	8 (R)	0.5 (S)	4 (I)	V595I	Q1392H	H501Q, S658L	WT
CP5689	Blood	8 (R)	0.5 (S)	4 (I)	V595I	Q1392H	S658L	WT
CP1387	Body fluid	2 (S)	0.5 (S)	2 (S)	WT	WT	S658L	WT
CP4632	Blood	1 (S)	1 (S)	1 (S)	WT	WT	S658L	WT

### Clinical data analysis

Fifty-four patients were diagnosed with candidemia and invasive candidiasis. The great majority (77.8%) of these had candidemia, and 12 (22.2%) had invasive candidiasis. Table 4 shows that their median age was 55.5 years (IQR 32, 69.8) and half were male. Fifty-seven percent of patients were admitted in the general ward, and 37% were in the intensive care unit. Approximately two-thirds had underlying diseases, notably diabetes mellitus (24.1%), solid or haematological malignancy (20.4% and 14.8%, respectively), and a minority (1.9%) with chronic haemodialysis. The notable risk factors for candida infection were prior broad-spectrum antibiotic therapy (98.1%), central venous catheterisation (66.7%), assisted ventilation (66.7%), indwelling urethral catheter (50%), and colonisation with candida (50%). The single clinical factor associated with fluconazole-resistant candidemia was duration of hospital stay. The median time of onset of candidemia in the fluconazole-resistant group was 34 days (IQR 30.5, 107), which was significantly longer than the 16 days (IQR 2, 28) in the fluconazole susceptible and fluconazole dose-dependent groups (*p* = 0.004). Only four patients (7.4%) had received fluconazole prior to hospitalisation. Amphotericin B (40.7%) was the most common antifungal agent prescribed for empirical treatment, followed by fluconazole (31.5%), and echinocandin (13%). The overall mortality rate was 48.1%, and there was no significant difference between the two groups (*p* = 0.14).

**Table 4.** Baseline characteristic of patients infected with fluconazole susceptible and susceptible dose-dependent and fluconazole-resistant candida, treatment, and outcomes

Clinical characteristics	Total (%)	Fluconazole-susceptible and susceptible dose dependent (SDD) candida^a^	Fluconazole-resistant candida^b^	*P*-value
No	54	42	12	
Age (years),	55.5	54	55.5	0.68
Median (IQR)	(32, 69.8)	(4.2, 73.5)	(32, 69.8)	
Male	28 (51.9)	23 (54.8)	5 (41.7)	0.64
Ward				0.2
Intensive care unit	20 (37)	16 (38.1)	4 (33.3)	
Semi intensive care unit	3 (5.6)	1 (2.4)	2 (16.7)	
General ward	31 (57.4)	25 (59.5)	6 (50)	
Underlying disease				
Solid malignancy	11 (20.4)	9 (21.4)	2 (16.7)	1.0
Haematologic malignancy	8 (14.8)	6 (14.3)	2 (16.7)	1.0
Diabetes mellitus	13 (24.1)	9 (21.4)	4 (33.3)	0.45
Chronic haemodialysis	1 (1.9)	1 (2.4)	0 (0)	1.0
Associated risk factor				
Prior received broad spectrum antibiotic	53 (98.1)	41 (97.6)	12 (100)	1.0
Steroid use	11 (20.4)	8 (19)	3 (25)	0.69
Indwelling urethral catheter	27 (50)	19 (45.2)	8 (66.7)	0.33
Central venous catheterisation	36 (66.7)	30 (71.4)	6 (50)	0.18
Parenteral nutrition	15 (27.8)	12 (28.6)	3 (25)	1
Recent abdominal surgery	11 (20.4)	9 (21.4)	2 (16.7)	1
Assisted ventilation	36 (66.7)	27 (64.3)	9 (75)	0.73
Neutropenia	11 (20.4)	6 (14.3)	5 (41.7)	0.05
Chemotherapy	14 (25.9)	9 (21.4)	5 (41.7)	0.26
Candida colonisation	27 (50)	22 (52.4)	5 (41.7)	0.74
Type of candida infection				0.30
Candidemia	38 (70.4)	31 (73.8)	7 (58.3)	
Invasive candidiasis	16 (29.6)	11 (26.2)	5 (41.7)	
Urinary tract infection	11 (20.4)	6 (14.3)	5 (41.7)	
Intraabdominal infection	3 (5.6)	3 (7.1)		
Osteomyelitis	1 (1.9)	1 (2.4)		
Pleural infection	1 (1.9)	1 (2.4)		
APACHE II score, mean (range)	18.4 (7.3)	18.6 (7)	17.7 (8.7)	0.75
Time onset of candidemia after	20.5	16	34	0.004
hospitalisation (days), median (IQR)	(3, 29.5)	(2, 28)	(30.5, 107)	
Prior received fluconazole	4 (7.4)	2 (4.8)	2 (16.7)	0.21
Empiric antifungal treatment				0.95
Amphotericin B (AmB)	22 (40.7)	16 (38.1)	6 (50)	
AmB and echinocandin	1 (1.9)	1 (2.4)	0 (0)	
Echinocandin	7 (13)	6 (14.3)	1 (8.3)	
Fluconazole	17 (31.5)	13 (31)	4 (33.3)	
No empirical treatment	7 (13)	6 (14.3)	1 (8.3)	
Overall in-hospital mortality	26 (48.1)	23 (54.8)	3 (25)	0.14
Mortality associated with candida infection	14/26 (53.8)	11/23 (47.8)	3/3 (100)	0.23

APACHE II, Acute Physiology and Chronic Health Evaluation II; IQR, interquartile range.

a Fluconazole SDD strains were *C. glabata* (*n* = 7), *C. tropicalis* (*n* = 4), and *C. parapsilosis* (*n* = 2).

b Fluconazole resistant strains were *C. tropicalis* (*n* = 8), *C. albicans* (*n* = 2), *C. parapsilosis* (*n* = 1), and *C. krusei* (*n* = 1).

## Discussion and conclusion

According to hospital-based data collected by Pfaller *et al*. [30] from 2006 to 2016, from Asia-Pacific participating countries of the SENTRY antifungal surveillance programme, *C. albicans* accounted for 46% of the 1,314 invasive isolates of *Candida* species. The distribution for the other species was *C. glabrata* (17.9%), *C. parapsilosis* (12.9%), *C. tropicalis* (14.1%), and *C. krusei* (1.8%) [30]. Our findings support the latter report and are in accord with other population-based surveys from Asia that *C. tropicalis*, *C. glabrata*, and *C. parapsilosis* were the most prominent of non-albicans *Candida* [31–33]. The shift of dominant species from *C. albicans* to non-albicans *Candida* has been observed since 2013. The rank order of the most common non-albicans in Thailand found in our study (Table 1 and Supplementary Table S1) was *C. tropicalis* (30.7%–39.1%) > *C. glabrata* (10%–22.22%) > *C. parapsilosis* (5.1%–19%). Candidemia due to *C. albicans* was the most common yeast infection (33%–38%) in keeping with varying data from other geographical areas such as the United States, Latin America, and China followed by *C. parapsilosis* (14%–26.5%), *C. tropicalis* (7%–17.6%), and *C. glabrata* (12.0%–30%) [34–36].

The prevalence of fluconazole-resistant candida documented here supports observations in worldwide reports from the SENTRY programme during the COVID-19 period where the species distribution of such isolates in rank order was *C. parapsilosis* (13.9%), *C. tropicalis* (3.5%), *C. glabrata* (2.0%), and *C. albicans* (0.1%) [20]. However, it is notable that *C. glabrata* reported in Thailand [37] and in our study displayed all isolates of this species exhibited susceptible-dose dependency with MIC values ranging from 2 to 32 μg/ml. We also noted a shift of MIC_50_ and MIC_90_ values of fluconazole in for *C. tropicalis* within the two periods, from 128 μg/ml in the pre-COVID-19 to 256 μg/ml in the pandemic. By contrast, *C. glabrata,* which is usually characterised by relatively low susceptibility to the azole drugs [38], displayed lower MIC_90_ values at 32 μg/ml. In China, the susceptibility of *C. tropicalis* to fluconazole also decreased to 57.5% in [36]. Unlike for *C. glabrata*, clinical awareness of using empirical fluconazole treatment for other *Candida* species and consequently fluconazole resistance has increased dramatically in *C. tropicalis, C. parapsilosis*, and *C. albicans* in Thailand [37]. As a result, infectious disease specialists have suggested that for severe candidemia cases, amphotericin B or echinocandin would be more appropriate than fluconazole [37].

Recent studies performed in several countries indicate the emergence of azole-resistant *C. tropicalis* isolates in clinical settings [30, 39–41]. In this study, we recorded an increase in the distribution of this species causing opportunistic and nosocomial infection, while its fluconazole susceptibility had almost halved (84.6% in pre-COVID-19 period to 41.7% in the COVID-19 period), even though isolates from 1997 to 2017 were generally fully susceptible to antifungal agents. This observation is corroborated by Wang *et al*. in 2021, on a large number of *C. tropicalis* isolates. They found that resistance rates of *C. tropicalis* to three azoles had increased year by year, with the fluconazole resistance rate reaching almost 40% [42]. Due to mild adverse reactions of azole drugs, and *C. tropicalis* being generally considered an azole-susceptible non-albicans species, fluconazole is the most widely used antifungal to treat patients’ infections with this species. However, long-term use of azoles might exert a selective pressure to select the emergence of azole-resistant strains [30].

The key statistically significant risk factor for the emergence of fluconazole-resistant strains in our study was time to onset of developing candidemia during hospitalisation as evidenced by the association of emergence of azole resistance in isolates from patients who had longer hospitalisation (34 vs. 16 days). A possible explanation could be the higher chance of antibiotic exposure with prolonged hospital stay, as almost all of such patients had received prior broad-spectrum antibiotic treatment and the definitive risk factor for fluconazole-resistant strains was prior receipt of fluconazole (7.4%). Ben-Ami *et al*. [43] reported that fluconazole-resistant candida isolates are associated with exposure to antibiotics, notably, carbapenems, colistin, trimethoprim-sulfamethoxazole, and clindamycin. The suggested potential underlying mechanism is the alteration of the gut microbiome by these agents which promotes gastrointestinal colonisation with drug-resistant *Candida* spp. [43]. However, examining the risk factors for acquisition of drug-resistant strains was outside the scope of our investigation.

The results of this study had a noticeable impact on clinical practice regarding the treatment of invasive *Candida* spp. Prior to this study, amphotericin B was the initial treatment of choice before species identification results were available, and fluconazole was then often prescribed if *C. albicans* was identified. However, our results showed that the percentage of fluconazole susceptibility of *C. albicans* and *C. tropicalis* markedly declined. As a consequence, AFST is now recommended to be performed for all candida isolates from sterile sites. Amphotericin B and echinocandin are now the recommended agents for treatment while awaiting susceptibility testing results. It is also notable that in our hospital, there is no currently prescribed antibiogram of antifungal drugs for *Candida* species, and therefore we recommend that such a guide should be generated annually in order to monitor resistance profiles and guide physicians to select the most appropriate antifungal agent [44].

Rates of resistance to echinocandin among *Candida* species were very low. Micafungin-resistant strains of *C. glabrata* reported worldwide through the SENTRY programme during the pre-COVID-19 and the COVID-19 periods were characterised by the rates of 1.7% and 2.0%, respectively [20]. In contrast, echinocandin-resistant *C. glabrata* (21.3%) and anidulafungin-resistant *C. parapsilosis* (16.7%) were observed in pre-COVID-19 time in our study. The most frequent alteration in HS regions of the 1,3-β-D-GS-encoding genes was an *FKS2* HS1 alteration S663P. This mutation was frequently displayed in echinocandin-resistant *C. glabrata* isolates from the United States, Germany, Spain, and Australia [20, 28, 30]. Uncommon echinocandin-resistant *C. parapsilosis* was documented in our study, and in a patient undergoing prolonged echinocandin therapy in Greece [45]. Novel alterations were detected in *FKS1* HS1 (V595I) and *FKS1* HS2 (Q1392H). Although never reported previously, these mutations were present in both isolates of anidulafungin-resistant *C. parapsilosis* in this study, and which were also non-susceptible to micafungin (MIC value, 4 μg/ml). The alteration in *FKS1* HS1 (F652S) was previously documented in pan-echinocandin resistant *C. parapsilosis* [46].

We acknowledge that this study was conducted with a relatively small number of candida isolates during COVID-19, particularly *C. glabrata* and uncommon *Candida* sp. As mentioned in Table 4, surgery and long-term hospitalisation are risk factors associated with candida infection including candidemia, catheter-associated UTI, catheter-associated bloodstream infection, and intrabdominal infection. However, the pandemic had a broad impact on surgical practice and case prioritisation [47]. Surgeries in our hospital were performed only in emergency cases, whereas elective and non-urgent surgeries were postponed. The consequent sharp decline of candida infection cases affected the number of isolates available for this study. Further limitations arose regarding the investigation of *FKS* mutation in one of the mechanisms exhibited in the antifungal-resistant candida. Although several mechanisms are operative among drug-resistant candida, alterations of target enzyme and efflux pump genes were not investigated for the increase of azole MICs. Another limitation of the study is that treatment outcomes were not determined, particularly for isolates that displayed reduced susceptibility to triazoles and echinocandins. Furthermore, antifungal agent exposure as prophylaxis or treatment was not investigated as a possible risk factor for drug-resistance during the COVID-19 period causing the MIC shift [45]. In addition, only a limited number of hospitals are capable of AFST in Thailand. Our study, conducted in one such hospital, produced results consistent with previously reported data from other tertiary hospitals in Thailand, particularly regarding *Candida* species distribution and the prevalence of antifungal drug resistance (Supplementary Table S1). Future large studies spanning multiple nations, and controlling for local factors, could be better indicators of any potential regional or global trend.

Continuing shifts in MIC value and antifungal drug resistance found here highlight the need for improved management of invasive candidiasis. To corroborate this finding, rapid and accurate identification methods to detect gene mutations associated with antifungal-drug resistance, and standardised susceptibility testing are essential for optimal patient care and management of bloodstream and deep-seated tissue candida infection. Current treatment guidelines recommend that early empirical and prophylactic therapy be initiated in high-risk individuals in the absence of an active infection, or prior to culture diagnosis [38, 48]. However, this non-specific implementation may lead to inappropriate use of some valuable antifungals in any instance, which is thought to be a key driver of emergent resistance. Thus, a promising direction for future research would be to further advance rapid, species-specific identification and drug-resistant candida infection detection methods, to effectively increase the prompt diagnosis of invasive infections. This would enable earlier initiation of antifungal therapy with a specific drug particularly for high-risk patients.

Regarding global policy-related challenges, the 2017–2021 WHO country cooperation strategy for Thailand established antimicrobial resistance as a priority area, leveraging the social and intellectual capital of partner countries in developing the required monitoring and evaluation systems, and generating the evidence needed to support the national strategic plan on antimicrobial resistance [49]. Academia and university hospitals are among the multistakeholder steering panels for the strategic plan, tasked with establishing an antimicrobial resistance surveillance system under WHO’s One Health approach. Its main challenge lies in the fragmented nature of the current multiple uncoordinated surveillance systems, many of which tend to focus on hospital-acquired infection and antibacterial resistance. As a result, a uniform surveillance system for antifungal resistance has yet to be fully established in Thailand. In this context, we have made a surprising discovery: the five Thai tertiary hospitals that perform AFST (Supplementary Table S1), including our hospital, all use similar systems for susceptibility determination and interpretation. Therefore, the usual challenges of data synchronisation and comparison of antifungal susceptibility profiles (such as MIC value and clinical breakpoint interpretation) between each hospital can be mitigated in this case, greatly facilitating the establishment of a uniform surveillance system for antifungal resistance. Another challenge lies in the lack of established national guidelines for the prevention and control of antifungal-drug resistance in human pathogenic fungi. Our findings of emerging drug-resistant candida in hospitals reinforce the urgent need for antifungal stewardship implementation in clinical settings, combined with effective education to foster appropriate antifungal use and decrease the incidence of drug resistant/non-susceptible candida. This is corroborated by Apisarnthanarak *et al*. [44] who demonstrated that the regular education of appropriateness of antimicrobial use for physicians and relevant healthcare staff, along with a well-planned antifungal stewardship programme for candidiasis, showed a significant decrease of inappropriate antifungal use at Thammasat University Hospital in Thailand [44].

In summary, we have presented a recent prospective observational study documenting the *Candida* species distribution, MIC distribution, and antifungal susceptibilities of invasive isolates over a 5-year period (pre- and during COVID-19) at a university hospital in southern Thailand. The detected shift of MIC value and increased prevalence of azole- and echinocandin-resistant in candida provide a strong warning of inappropriate use of fluconazole as an empirical treatment. Therefore, *in vitro* AFST of suspected fungal pathogens is necessary for laboratory surveillance of antifungal resistance in candida, and for providing valuable data for a more effective infection control in the hospital.

## Supporting information

Szekely et al. supplementary material Szekely et al. supplementary material

## Data Availability

Data supporting reported results may be provided on reasonable request to the corresponding authors.

## Supplementary material

For supplementary material accompanying this paper visit http://doi.org/10.1017/S0950268823001346. click here to view supplementary material
