# Supplementary material for: Fluconazole and echinocandin resistance of Candida species in invasive candidiasis at a university hospital during pre-COVID-19 and the COVID-19 outbreak
Source: Epidemiol Infect. 2023 Aug 25;151:e146. doi: 10.1017/S0950268823001346 (PMC10540169; doi:10.1017/S0950268823001346)
Supplement: Szekely et al. supplementary material [file S0950268823001346sup001.docx]

Supplementary Table S1. *Candida* species distribution from 1997 to 2021 in Thailand and countries in the Asia-Pacific Region

| **Organism** | **Number of *Candida* isolates (%)** | | | | | | |
| --- | --- | --- | --- | --- | --- | --- | --- |
|  | **1997-2000**  **(Thailand) [1]** | **2012-2013**  **(Thailand) [2]** | **2013-2015**  **(****Asia-Pacific**  **Region*) [3]** | **2015**  **(Thailand) [4]** | **2016-2017**  **(Thailand)**  **[5]** | **2018-2019**  **(Thailand)**  **[6]** | **2017-2021**  **(This study)** |
| *Candida albicans*  Non-albicans Candida  *C. tropicalis*  *C. parapsilosis*  *C. metapsilosis*  *C. orthopsilosis*  *C. guilliermondii*  *C. glabrata*  *C. nivariensis*  *C. norvegenesis*  *C. dubliniensis*  *C. duobushaemulonii*  *C. caribbica*  *C. fabianii*  *C. famata*  *C. haemulonii*  *C. intermedia*  *C. krusei*  *C. lusitaniae*  *C. pelliculosa*  *C. pseudohaemulonii*  *C. rugosa* | 101 (42.9%)  13 (9.9%)  2 (1.52%)  -  -  1 (0.76%)  -  -  -  -  -  -  -  -  -  -  -  -  -  -  - | 35 (53%)  18 (27.3%)  3 (4.5%)  -  -  -  10 (15.2%)  -  -  -  -  -  -  -  -  -  -  -  -  -  - | 309 (35.9%)  264 (30.7%)  135 (15.7%)  7 (0.8%)  116 (13.6%)  1 (0.1%)  1 (0.1%)  3 (0.3%)  -  -  1 (0.1%)  1 (0.1%)  1 (0.1%)  1 (0.1%)  6 (0.7%)  1 (0.1%)  6 (0.7%)  1 (0.1%)  6 (0.7%) | 19 (36.5%)  18 (35%)  10 (19%)  -  -  -  5 (10%)  -  -  -  -  -  -  -  -  -  -  -  -  -  - | 45 (28.8%)  77 (49.4%)  8 (5.1%)  -  -  -  26 (16.7%)  -  -  -  -  -  -  -  -  -  -  -  -  -  - | 16 (29.63%)  18 (33.33%)  5 (9.26%)  -  -  1 (1.85%)  12 (22.22%)  1 (1.85%)  -  -  1 (1.85%)  -  -  -  -  -  -  -  -  -  - | 33 (24.8%)  52 (39.1%)  18 (13.5%)  7 (5.3%)  2 (1.5%)  1 (0.75%)  14 (10.5%)  1 (0.75%)  -  1 (0.75%)  1 (0.75%)  1 (0.75%)  -  -  1 (0.75%)  -  1 (0.75%)  -  -  -  - |

* The countries in study [3] include Brunei, Korea, Philippines, Singapore, Taiwan, Thailand, and Vietnam.

**References**

1. **Tritipwanit K, Chindamporn A, Suankratay C**. (2005) Epidemiology of candidemia at King Chulalongkorn Memorial Hospital, Thailand. *Journal of Infectious Diseases and Antimicrobial Agents*; **22**: 59–69.

2. **Thunyaharn S, *et al.*** (2021) Activity of triazoles and echinocandins against *Candida* bloodstream isolates at Phramongkutklao hospital, Thailand. *Journal of Southeast Asian Medical Research*; **5**: 84–90.

3. **Tan BH, *et al.*** (2005) Incidence and species distribution of candidaemia in Asia: a laboratory-based surveillance study. *Clinical Microbiology and Infection*; **21**: 946–953.

4. **Naranong C, Anunnatsiri S, Srigulbutr S**. (2020) Epidemiology and antifungal susceptibility in patients with candidemia in a university hospital, Thailand. *Journal of the Medical Association of Thailand*; **103**: 1048–1056.

5. **Pham LTT, *et al.*** (2019) A predominance of clade 17 *Candida albicans* isolated from hemocultures in a tertiary care hospital in Thailand. *Frontiers in Microbiology*; **10**: 1194.

6. **Boonsilp S, *et al.*** (2021) Species distribution, antifungal susceptibility, and molecular epidemiology of *Candida* species causing candidemia in a tertiary care hospital in Bangkok, Thailand. *Journal of Fungi*; **7**: 577.
